# Gradenigo’s Syndrome With Septic Lateral Sinus Thrombosis

**DOI:** 10.7759/cureus.34797

**Published:** 2023-02-09

**Authors:** Tiago Branco, Catarina Marques, Vera C Santos, João M Lopes

**Affiliations:** 1 Serviço de Medicina 2, Hospital de Santa Maria, Centro Hospitalar Universitário Lisboa Norte, Lisboa, PRT; 2 Internal Medicine, Hospital José Joaquim Fernandes, Beja, PRT; 3 Serviço de Medicina Intensiva, Hospital Prof. Doutor Fernando Fonseca, Amadora, PRT

**Keywords:** gradenigo’s syndrome, chronic suppurative otitis media, septic thrombosis, otitis media complication, lateral sinus thrombosis

## Abstract

Gradenigo’s syndrome (GS) is a rare but life-threatening complication of acute otitis media (AOM). It is classically defined as a clinical triad of acute otitis media, ipsilateral sixth (abducens) nerve palsy, and pain in the distribution of the first and second branches of the trigeminal nerve. Another rare but serious complication of AOM is venous sinus thrombosis, which is often associated with GS. The diagnosis of these conditions requires clinical suspicion, sound interpretation of signs and symptoms, and the use of the correct imaging techniques.

Here, we present the case of an 81-year-old man with a previous history of recurrent otitis media, who presented with GS and septic lateral sinus thrombosis. The clinical presentation, physiopathology, and management of these conditions are discussed.

## Introduction

Gradenigo’s syndrome (GS) is a clinical triad of acute otitis media (AOM), ipsilateral sixth (abducens) nerve palsy, and pain in the distribution of the first and second branches of the trigeminal nerve, first described by Professor Giuseppe Gradenigo in 1904 [1]. Classically, these symptoms are assumed to be a consequence of the inflammation of the petrous apex of the temporal bone and Dorello’s canal, a condition termed apical petrositis (or petrous apicitis) usually caused by medial extension of AOM into a pneumatized apex located near the trigeminal ganglion and sixth cranial nerve [2]. After the introduction of antibiotics in the early 20th century, the incidence of GS has diminished, and is now considered a rare, but still life-threatening condition [3]. It is more frequent in adults [4]. The incidence of apical petrositis is reported to be two per 100,000 children with AOM [5].

GS can be associated with other complications, such as meningitis and venous sinus thrombosis [6]. Septic venous sinus (lateral, cavernous, or sagittal) thrombosis is also a serious complication of AOM with high mortality, particularly in elderly patients [7,8]. Chronic or untreated AOM may result in the spreading of the infection to the mastoid air cell, leading to thrombosis of the lateral sinus, which may then propagate to the internal jugular vein and other dural sinuses [9]. The infection usually reflects the polymicrobial nature of otitis media. Physical findings may include fever, which is less prominent in those associated with chronic otitis media [8], and, when present, GS provides strong evidence for lateral sinus thrombosis or petrous apex inflammation [10]. Prompt administration of large-spectrum antibiotics is paramount, and in the absence of rapid defervescence, surgery is indicated [8].

## Case presentation

An 81-year-old man with a previous history of hypertension, cerebrovascular disease, and type 2 diabetes was admitted to the emergency department for a three-week course of nausea, vomiting, headache, and ataxia. The headache was described as affecting mainly the left parietal and temporal regions, with mention to left retro-orbital pain. Investigation of clinical history revealed recurring otitis media infections with previous complaints of headache, vertigo, and ataxia along with left imbalance for the last six months.

**Figure 1:**
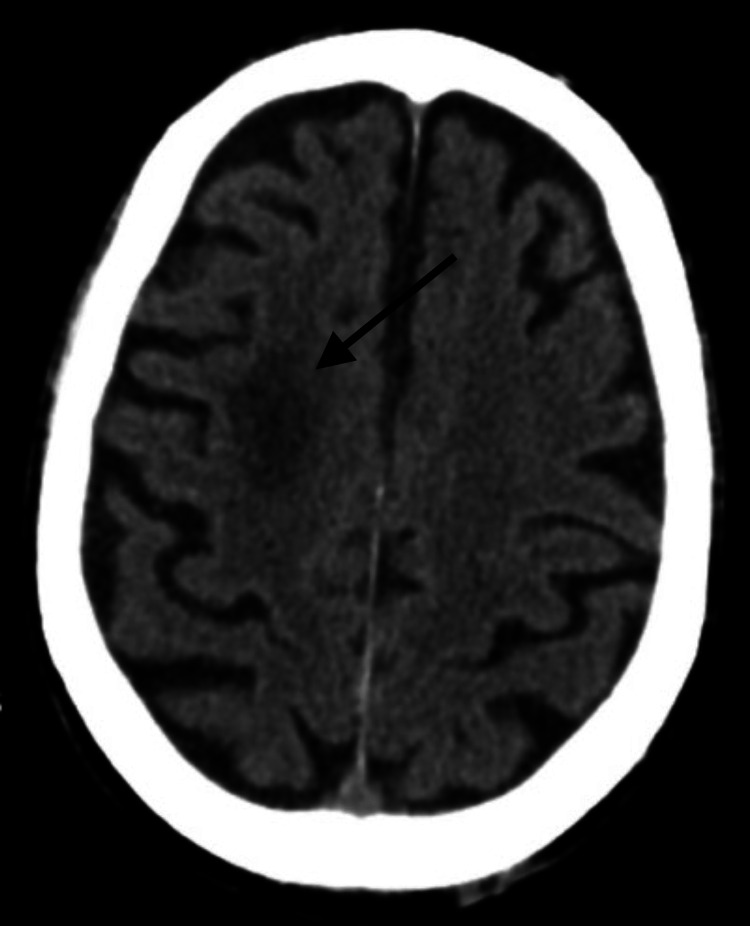
Head CT showing ischemic lesion sequelae.

**Figure 2:**
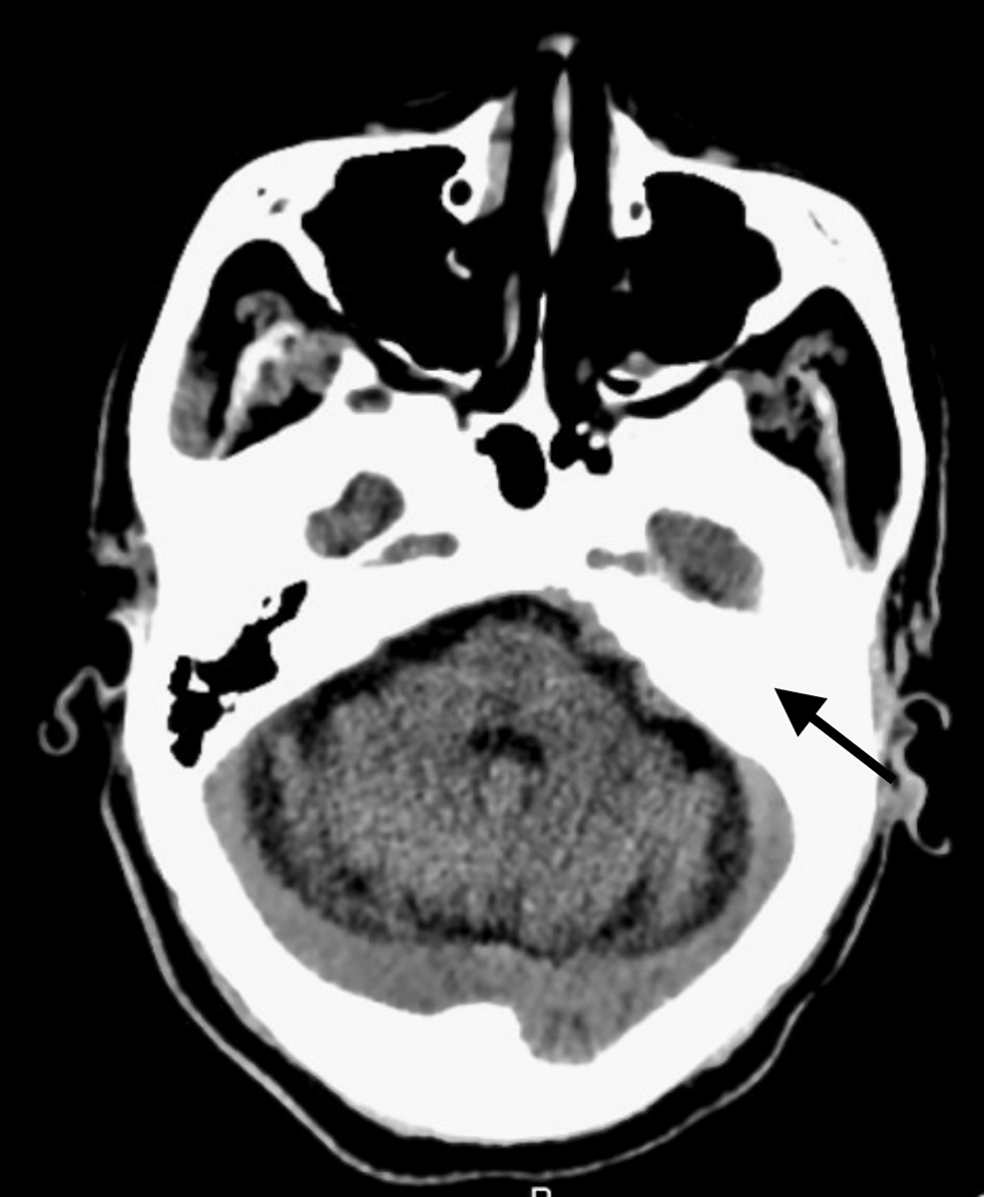
Head CT showing left middle ear and mastoid filling.

On physical examination, he presented with left hearing loss, peripheral vertigo, left gait ataxia, and left external oculomotor ophthalmoplegia, with no meningeal signs. A slightly elevated C-reactive protein (CRP) (4.4 mg/dL; normal <0.9 mg/dL) was the only relevant finding on blood tests. Diabetes was reasonably controlled (HbA1c 7.6%). Head CT showed only ischemic lesion sequelae and left middle ear and mastoid filling (Figures 1, 2).

**Figure 3:**
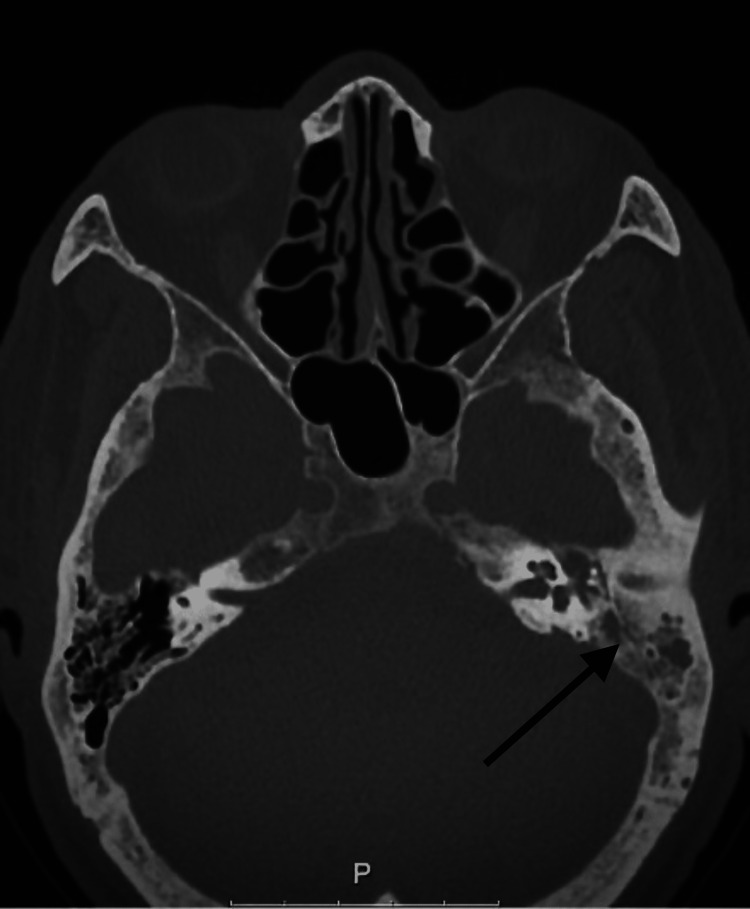
Ear CT revealing skull base osteomyelitis and left otomastoiditis.

Upon admission to the ward, blood cultures were drawn and empirical antibiotherapy was started with intravenous (IV) ceftriaxone 2 g every 12 hours. Further investigation with ear CT revealed skull base osteomyelitis and left otomastoiditis (Figure 3).

Additional findings of infectious collections in the cerebellopontine angle and retropharyngeal space were also documented. Antibiotherapy was then changed to meropenem (2 g every eight hours) to improve antibiotic penetration and add coverage to *Pseudomonas aeruginosa*.

Neurology and neurosurgery opted for a conservative approach and discarded surgical control of the cerebellopontine and retropharyngeal collections.

**Figure 4:**
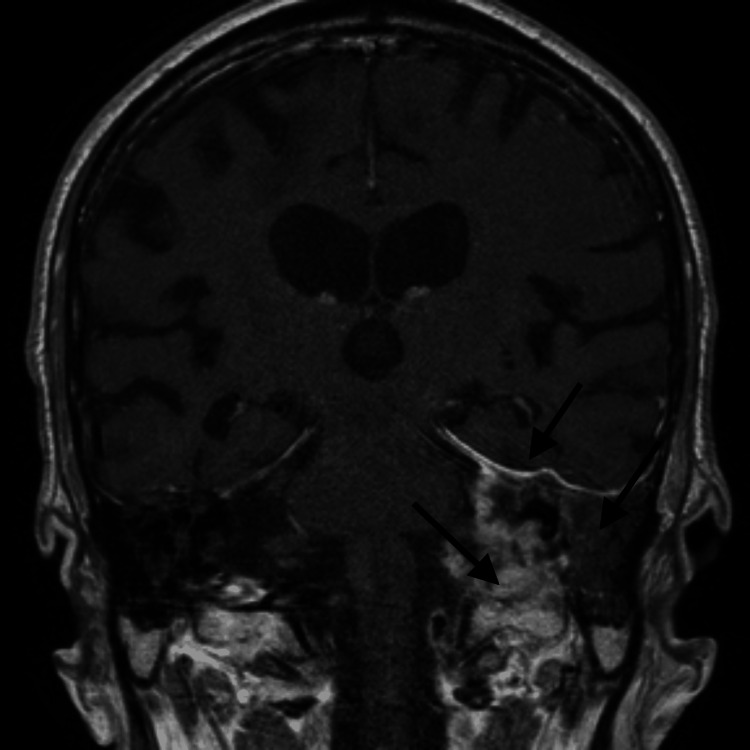
T2-weighted head MRI documenting left otomastoiditis with pachymeningeal inflammation.

**Figure 5:**
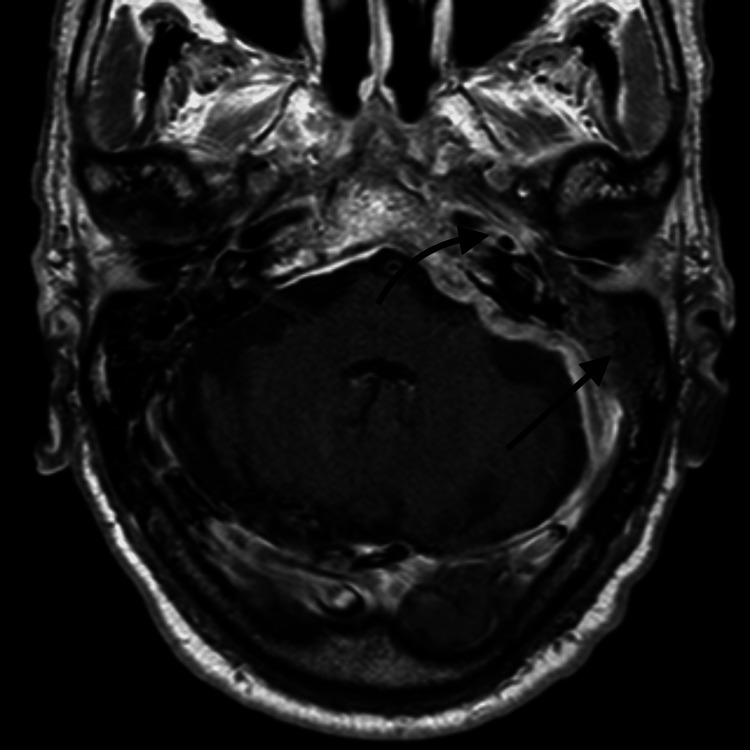
T1-weighted MRI with gadolinium documenting septic left lateral sinus thrombosis with apical petrositis.

The case was then discussed with the otolaryngology department and surgical intervention was decided. Prior to the surgery, a head MRI confirmed left otomastoiditis, labyrinthitis, and documented septic left lateral sinus thrombosis with pachymeningeal inflammation (Figures 4, 5).

It was decided not to start anticoagulation at this point, considering that it could increase hemorrhagic risk and hematogenous infection dissemination.

The patient was then submitted to exploratory tympanostomy, closed technique mastoidectomy, and myringotomy with Shepard tympanostomy tube insertion in the left ear. During the procedure, samples were collected for cytochemical, bacterial, mycobacterial, and anatomopathological studies. Samples were also collected for 16s rRNA gene sequencing. No microorganism was isolated or identified in any of the samples.

The patient then began a long recovery process adjuvanted by physical therapy. At this point, anticoagulation with enoxaparin in a prophylactic dose was started. Only partial symptomatic recovery was achieved, with persistent sixth nerve palsy two months after the surgery. Subsequent MRI studies revealed the persistence of the infectious cerebellopontine and retropharyngeal collections and lateral sinus thrombosis. The need for long-term IV antibiotics (at least six months) conditioned discharge, but the patient was eventually able to go back home under a domestic hospitalization protocol. Three months later, he suffered a fatal ischemic stroke, affecting the right precentral gyrus. Although the lesion was consistent with an embolic etiology, the relationship of this event with lateral sinus thrombosis remains unclear.

## Discussion

Septic lateral sinus thrombosis is a rare complication of otitis media, and GS is an exceedingly rare presentation of otitis media complications. The time interval between the onset of otitis media and the clinical presentation of Gradenigo’s triad of symptoms is variable. Despite sharing otitis media as the common etiology, each symptom results from different physiopathological processes. In a 40-year review of apical petrositis cases, only 13.6% presented all the GS triad’s symptoms [4]. A recent review of all reported cases over the last 50 years found only 45 cases of classic GS presentation and proposed a new classification of incomplete GS and GS mimicking other presentations of apical petrositis [11].

In general, the petrous apex is composed of dense bone and bone marrow. In only one-third of the cases, petrous apexes are well pneumatized and communicate with the middle ear cleft [12], making it more susceptible to obstruction, opacification, inflammation, and infection. This is the same process by which the pneumatized mastoid segment is also vulnerable to middle ear infection and the reason why it is usually associated with GS. However, GS can also occur on a background of previous radical mastoidectomy [2].

The petrous apex is located at the central part of the temporal bone. A fold of medial dura mater of the petrous apex forms Dorello’s canal, in which the sixth nerve rises toward the cavernous sinus [13]. The proximity of the Gasserian ganglion, from which the ophthalmic and maxillary branches of the trigeminal nerve arise, can cause AOM patients to experience retro-orbital pain. This symptom was present in 54.6% of the cases according to the review by Gadre and Chole [4] and can be one of the earliest signs of GS [14]. Development of abducens nerve palsy varies from one week to two to three months after the initial infection [10] and is present in only 15.9% of patients with apical petrositis [4]. The average time to presentation from symptom onset for classic GS is 14.9 days [11].

In the present case, ear CT revealed skull base osteomyelitis and left otomastoiditis, triggering further investigation with MRI. Although CT scans are adequate for describing bone anatomy and bone destruction, other methods are preferred for diagnosing central nervous system pathologies and the composition of the lesion in the bone. In a series of 49 patients, head CT failed to identify 14% of the patients with central venous thrombosis, whereas venous CT or MRI venography could provide positive identification of abnormalities in all cases [15]. Clinical presentation may be atypical and high clinical suspicion may be needed to prompt image investigation [16]. In cases of infectious petrositis, MRI shows hypointensity on T1-weighted and hyperintensity on T2-weighted images. In cases of abscess formation, a rim of gadolinium enhancement and diffusion-weighted imaging hyperintensity may be observed [14].

Identification of infectious agents was not possible in the presented case, but recent reviews found the microbiology of the AOM infections leading to GS surprising, for the preponderance of aerobic gram-positive cocci, and the relative paucity of *Streptococcus pneumonia* and *Haemophilus influenza*, common pathogens in otitis media and mastoiditis [11]. Infectious petrositis is equivalent to osteomyelitis, which needs intensive and prolonged IV antibiotic treatment to avoid relapse [14].

Many cases of successful conservative treatment with IV antibiotics have been reported. However, if the patient deteriorates, or in the absence of rapid improvement, immediate surgery is indicated [4,14]. This approach, when considered, should remove as much of the infected temporal bone as possible with preservation of hearing and facial nerve functions, including mastoidectomy and the exposition of petrous apex by media fossa [13].

The benefit of anticoagulation treatment in this condition is debatable and remains controversial. In a series of five cases involving septic lateral sinus thrombosis under long-term anticoagulation with low-molecular-weight heparin, there was only one reported complication of a non-life-threatening intracranial hemorrhage [17]. A Cochrane review designed to assess the effectiveness and safety of anticoagulant therapy in patients with confirmed non-septic cerebral venous sinus thrombosis suggested that anticoagulant drugs are probably safe and may be beneficial for people with sinus thrombosis, with a potentially important reduction in the risk of death or dependency. The results, however, did not reach statistical significance due to the limited evidence available [18]. In the present case, after consultation with several specialties, it was decided not to introduce therapeutic anticoagulation, which may have contributed to a fatal outcome.

## Conclusions

GS and lateral sinus thrombosis are rare but life-threatening complications of acute and chronic otitis media. Clinical interpretation of the signs and symptoms can be challenging as the time interval between the onset of otitis media and the clinical presentation of Gradenigo’s triad of symptoms is variable, and not all patients present with the classical syndrome.

Imaging studies should include venous CT or MRI venography to exclude cerebral venous thrombosis. The use of anticoagulation treatment remains controversial but is probably safe and may be beneficial. Prompt treatment with large-spectrum IV antibiotics is paramount, and in the absence of rapid improvement, immediate surgery is indicated.

The presented case highlights the pivotal role of internal medicine in the management of a complex condition that required continuous consultation with several surgical and non-surgical specialties (neurology, neurosurgery, otolaryngology, neuroradiology, ophthalmology, and infectiology).
